# Editorial

**Published:** 1890-09

**Authors:** 


### Editorial.

### HOW TO LIVE TO BE THREESCORE AND TWENTY

Under this title, Dr. Oliver Wendell Holmes, himself an octogenarian, contributes an article to the July number of the *Atlantic Monthly*, and it is interesting to learn the steps that he advises, in order to attain the ripe old age which he so much adorns.

In the first place, where practical, he recommends that the parents be selected from a stock that attains to old age ; especially should the mother "come of a race in which octogenarians and nonagenarians are common phenomena." In the absence of the chance for exercising this selective prerogative good fortune may have arranged it for you.

Next he ranks *cheerfulness*, and lays down the rule, "Take all the troubles and trials of the world with perfect equanimity and a smiling countenance." To some, he says, this is no trouble, but with others it requires effort. "Your friend, the curly-haired blonde, with the lungs of a pearl diver and the stomach of an ostrich, the best possible digestion and respiration, finds it perfectly easy to carry them into practice; but you of leaden complexion, with black and lank hair, lean, hollow-eyed and dyspeptic, find it difficult." This will in all probability, he says, give you from three to five years.

The next advice is, as he predicts, a little startling. "One of my prescriptions is this: *Become a subject of a mortal disease.* Let half a dozen doctors thump you, and knead you, and test you in every possible way, and then render the verdict that you have an internal complaint; they don't know exactly what it is, but it will certainly kill you by and by. Then bid farewell to the world and shut yourself up for an invalid. If you are three score years when you begin this kind of life you may last twenty years, and there you are an octogenarian. In the meantime your friends have been dropping off one after another, until you find yourself almost alone, nursing your mortal complaint as if it were a baby, hugging it and kept alive by it—if to exist is to live. Persons who are shut up this way, confined to their chambers, sometimes to their beds, have a very small amount of vital expenditure, and wear out very little of their living substance. They are like lamps, with half of the wick picked down, and will continue to burn when other lamps have used up all their oil. Thus, he says, has Dr. Mitchell attained brilliant results by saving the vital expenditure of his patients.

Turning to the more practical part of the article, we find the question, "What have I got to say in reference to temperance, the use of animal food, &c.?" And in reply, "Nature has proved a wise teacher, as I think, in my own case. The older I grow, the less use I make of alchoholic stimulants. In fact, I hardly meddle with any of them, except an occasional glass of champagne. I find that by far the best borne of all drinks containing alcohol. I do not suppose that my experience can be the foundation for a universal rule. Dr. Holyoke, who lived to be a hundred years old, used habitually, in moderate quantities, a mixture of cider, water and rum. I think as we grow older less food of an animal nature is required."

To those who hold that the use of alcohol is necessary this will not be very glad tidings, but it was not essential that the venerable physician should say to the profession that the continued use of alcoholic stimulants can well be done away with. Though like St. Paul, they may take it for the stomach's sake, yet they may more often have to take something for the stomach as a result of too much alcohol in the earlier part of life.

Of another habit he says: "What do I say of smoking? I cannot grudge an old man his pipe, but I think tobacco does a great deal of harm to the health—to the eyes especially, to the nervous system generally, producing headache, palpitation and trembling. I myself gave it up many years ago. Philosophically speaking, I think self-narcotization and self-alcoholization are rather ignoble substitutes for undisturbed self-consciousness and unfettered self-control."

Lastly, after dwelling upon the use of oatmeal and such articles of diet, the doctor comes to the defense of the much abused American pie. "There is a very odd prejudice against pie as an article of diet. It is common to hear every form of bodily degeneracy and infirmity attributed to this particular favorite food. I see no reason for it. Mr. Emerson believed in pie, and was almost indignant when a fellow traveler refused the slice that was offered him. 'Why Mr. ———, said he, what is pie made for?' If every Green Mountain boy has not eaten a thousand times his weight in apple, pumpkin, squash and mince pie, call me a dumpling. And Colonel Ethan Allen was one of them—Ethan Allen, who, as they used to say, could wrench off the head of a wrought nail with his teeth."

While it may not be that by following the advice given in the quotations made we can attain this green old age, yet there is much that we might all well heed. In these days of rush and worry, of anxiety and excess, it would be for the good of many a man to follow the rules of him who has practically reached, and enjoyed, the threescore and twenty, and is yet hale and hearty, as well as being vigorous in mind. May we all even approach him in the successful performance of the feat.

---

### THE SIGNIFICATION OF SMALL DÓSES.

In view of the fact that there is a general and manifest tendency towards the employment of small doses, it is well to remember, lest we also be thought to be imitating the homeopaths, that the latter possesses no peculiar claims upon this method of administering medicines. Homeopathy bases its pretentions for notoriety solely upon their so-called law which is expressed in the formula, *similia similibus curantur*, and has nothing at all to do with the amount of the drug, however much some of its advocates have gone to extremes in this matter. Indeed the discoverer and the originator of this doctrine possessed no fixed notion about dosage, and vacillated between both extremes, and even now very few of his representative successors are agreed upon the method of giving the remedy. Hence, small doses given singly and alone is not homeopathy, and has nothing peculiar to it, the distinguishing factor being a law for the selection of the remedy according to the symptomatology.

The fact is, no school or sect can claim to be the originator of this method of administering medicine. It is the combined result of a long series of observations, and the consequence of a general conservatism that is pervading all branches of our science. The homeopath gave small doses in accordance to an assumed law of therapeutics, we give small doses because reliable experience and observation demonstrate their utility.

B.

### ADVERTISING DOCTORS.

The July issue of the Atlanta *Medical and Surgical Journal* contains an opportune article on the subject of doctors that advertise in the secular press, or more properly speaking, those who allow their pictures, accompanied by a highly complimentary life sketch, to appear in those pages. While such things do at times occur without the knowledge or consent of the physician, yet it is strange that the "lightning" can so often strike in the same place.

The extent to which the newspapers get hold of the details of important operations that are performed in some quiet locality has also for an indefinite period been a matter of mystery.

While these things are to be deplored, and are justly condemned, we would call attention to the unethical advertising that appears in the medical press. We believe that the code has declared that the physician who confines his attention to a specialty can with propriety advertise that his practice is "limited" to such and such a class of diseases, but there is no authority to permit a man who is engaged in the general practice of medicine and surgery to insert in the pages of a medical jourual, "Dr. Smith, surgeon," when the same Dr. Smith is well pleased to receive a call to a case of typhoid fever or pneumonia, or to attend a case of obstetrics. Manifestly such conduct is in the extreme irregular, and subjects the advertiser to just criticism. Many good surgeons are good practitioners, and in the smaller cities it is comparatively rare that one has enough work to justify the exclusion of all medical cases. Then why should one man have the right to present his claims so boldly, while his more modest and less advertised colleague must content himself with making himself "known by his works." While we fully agree with our contemporary as far as the attack upon advertising doctors went, yet we would respectfully suggest that those in the medical press should curb the tendency that so oversteps the ethical line.

### TRI-STATE MEDICAL ASSOCIATION OF ALABAMA, GEORGIA AND TENNESSEE.

The second annual session of the Tri-State Medical Association will be held at Chattanooga, Tenn., October 14, 15 and 16.

The meeting promises to be a great success. Gentlemen desiring to read papers should send titles of the same to the Secretary, Dr. Frank Trester Smith, Chattanooga, Tenn., at an early date.

### THE MISSISSIPPI VALLEY MEDICAL ASSOCIATION.

The Mississippi Valley Medical Association is the outgrowth of the old Tri-State Medical Society, and of its many meetings the one to be held in Louisville in October promises to be the best. Medical men prominent in medicine in the Mississippi Valley have given their names to the Secretary for papers. Dr. John A. Wyeth, of New York, has consented to be present and read a paper, as has also Dr. Frank Woodbury, of Philadelphia. The Association devotes itself strictly to business, and no side issues are allowed. If a man has a grievance he is referred to committee. The President is Dr. Joseph M. Mathews, of Louisville; Secretary, Dr. E. S. McKee, Cincinnati; Chairman Committee of Arrangements, Dr. I. N. Bloom, Louisville.

### THE AMERICAN RHINOLOGICAL ASSOCIATION.

The American Rhinological Association will hold its Eighth Annual Session at Louisville, Ky., October 6, 7 and 8.

All leading subjects relating to nasal and naso-pharyngeal disease will be opened for discussion by a leading fellow of the Association. The medical profession is cordially invited to attend.

The Secretary, Dr. R. S. Knode, Omaha, Nebraska, will furnish any information to physicians desiring to become members.

---

SAND BAGS A CONVENIENCE.—The sand bag is invaluable in the sick room. Get some clean, fine sand, dry it thoroughly in a kettle on a stove. Make a bag, about eight inches square, of flannel, fill it with dry sand, sew the opening carefully, and cover the bag with cotton or linen. This will prevent the sand from sifting out, and will also enable you to heat the bag quickly by placing it in the oven or even on top of the stove. After once using this you will never again attempt to warm the feet or hands of a sick person with a bottle of hot water or a brick. The sand holds the heat a long time, and the bag can be tucked up to the back without hurting the invalid. It is a good plan to make two or three of the bags, and keep them on hand ready for use at any time when needed.—*The Nightingale.*

---

THE DRY METHOD OF TREATING WOUNDS.—The dry method of wound treatment is receiving considerable attention. Dr. Lauderer, of Leipzig, is one of the pioneers. No fluid is allowed to touch the wound. Dry aseptic gauze is used in place of moist sponges and irrigating fluids, and pieces of gauze are applied to the wound. He claims the following advantages:

First—The patient is not exposed to wet and cold.

Second—The loss of blood is minimal.

Third—Absorption of antiseptics is not possible.

Fourth—Time of operation is decreased.

Fifth—Rapid recovery, only one dressing being necessary, and that only if non-absorbable stiches are used.

Sixth—Saving of surgeon's hands.—*Gaillard's Medical Journal.*

